# Hôtel Dieu

**Published:** 1829-04-01

**Authors:** 


### Periscope;

OR,

### CIRCUMSPECTIVE REVIEW.

" Ore trahit quodcunque potest, atque addit acervo."

### CLINICAL REVIEW, N° IV.

### I.

### HOTEL DIEU.

I. Operation of Lithotomy—M. Dupuytren unable to extract the Stone.

Whilst the medical profession in this country has been fully occupied with the consideration of a case of lithotomy, an unfortunate operation of the same description has happened in the practice of M. Dupuytren at the Hôtel Dieu, and excited a considerable sensation in the surgical *salons* of Paris. The following are the particulars of the case; and the manner in which it is related by the French reporter forms a striking contrast with that which is adopted by the pitiful hirelings that pander to the appetite for scandal in England.

A man of fifty years of age, of small stature, and somewhat fat, who had long been affected with gravel, and often passed good-sized stones by the urethra, had, for ten years previous to his admission in the Hôtel Dieu, ceased to void sand in his urine, but experienced all the symptoms of stone in the bladder. The existence of a calculus was ascertained by several surgeons of Angers, and, on making an attempt to introduce the sound, M. Dupuytren found that its progress was arrested by a foreign body, immediately beyond the neck of the bladder. On placing the finger in the rectum, the bladder was felt to be distended by a hard and voluminous body, which was also felt on firmly pressing down the hypogastric region. The stone, from this examination, appeared to be more than two inches in length, and the patient was ready to submit to any operation for its removal. After balancing in his mind the various operations, M. Dupuytren determined on proceeding in the manner we are now to describe.

A nearly straight sound was placed in the urethra, and introduced as far as the stone would allow; a finger in the rectum distinguished the groove in the staff; and this being felt, the perineum and sphincter ani were divided, to the extent of an inch and a half, by the scalpel, which was carried along the groove of the instrument into the bladder. The section of this viscus was made, however, with M. Dupuytren's double lithotome, opened to the angle 15. The forceps were introduced, and, after some difficulty, the calculus was seized, but it could not be extracted. Several unsuccessful attempts at abstracting it were made, but at length M. Dupuytren desisted, and the patient was removed from the operating theatre, with the calculus still remaining in the bladder. He was placed in the bath for three quarters of an hour, and in the afternoon twenty-four leeches were applied to the hypogastrium and the bath repeated, with good effect. In the evening he was bled from the arm to two " palettes," which produced some nausea, and, during the night, the bath was repeated, for the third time. Next morning the pulse was somewhat fuller, and the passage of urine in the rectum gave pain. An anodyne was given, which procured some sleep, but the nausea continued, the tongue was white, and pain was experienced in the left iliac region, which symptoms continued unabated on the following day. Leeches were applied to the iliac region, and the patient complaining of a wish to go to stool, some doses of calomel, and plenty of " orangeade," were given, whilst fomentations were applied to the belly, and another bleeding was practised in the arm. In the evening there was pain in the perineum, and on passing the finger into the wound, the stone was felt to have descended. The lower part of the bladder was "vertically" divided by a probe-pointed bistoury, as well as the kind of buttress left at the anterior wall of the rectum, a pair of forceps were introduced into the bladder, the stone laid hold of, and, after some trouble, extracted. It was oval and flattened in shape, polished in some parts, rough in others, and weighed five ounces, thirty-six grains. Its length was two inches and nine lines, its breadth two inches and three lines, and its thickness an inch and eight lines. No hæmorrhage occurred, but the symptoms of depression made progress, and at one in the morning of the fourth day after the operation the patient died.

On dissection, there was found a trifling collection of pus in the cellular membrane uniting the rectum and bladder, the latter organ being thickened, and presenting traces of old ulcerations in its internal membrane. The kidneys contained a number of cysts, the size of a nut, which were filled with pus and a concrete matter, like adipocere.

The reporter of the case in the Clinique has made some critical remarks upon the operation, which, be they right or be they wrong, are at any rate candid, and free from that horrible slang and abuse which characterise such writings in this happy land. Our neighbours are behind us in this respect, for they yet must learn, that the quickest way to the pockets of the public, is personal invective, slander, and libel. The above operation is one illustration in a million, of the difficulties that often beset the lithotomist, that foil his skill, unnerve his arm, endanger the existence of his patient, and cover himself with obloquy and dishonour in the minds of the weak, or the hands of the malignant. Here we see a man who, by common consent, is considered one of the ablest surgeons in Europe, certainly one of the most dexterous operators, failing to extract the stone for which he cut. Had such a thing happened to a RAT or a CORRUPTIONIST here, what a tale of terror would, without all doubt, have been cooked and hashed up for the public stomach, what "butchery and blood," and other such choice and "professional" expressions, would have graced the pages of the RADICAL PRESS! By the bye, the case detailed above is a good example of the *benefits* of the calculus remaining in the bladder, for a second operation was actually and literally required to extract it. Two operations, as Sir Astley observes, are worse to bear than one.

II. CALCULUS IN THE BLADDER—LITHONTRIPTEUR TRIED WITHOUT SUCCESS—LITHOTOMY PERFORMED.

A little girl, eight years of age, was brought to the Hôtel Dieu, on the 4th of November, 1827, with the symptoms of stone in the bladder. On inquiry, it was found that the symptoms had existed for four years, and at times were so severe as to cause convulsions. A year and a half after their commencement, M. Baffos was consulted, discovered the stone, dilated the urethra, introduced the forceps, seized the stone, and broke it down with the greatest facility. The fragments came away with the urine, and the little patient was relieved from her former sufferings, although the dilatation of the urethra gave rise to an incontinence of urine. This relief continued for nearly three years; but in April, 1827, she entered the Ecole de Médecine, with all the symptoms of a return of the disease, when M. Breschet several times introduced the forceps, and drew out some portions of calculus, similar to those which had formerly been discharged. The symptoms, however, still continued, and the parents took their child to Messrs. Wesely and Civiale, who tried six different times to break down the stone, but occasioned such pain, that they were absolutely forced to discontinue their attempts. It was shortly after this that the patient was brought to the Hôtel Dieu, and M. Dupuytren having fully convinced himself of the actual presence of the stone, and remarked the pain produced by sounding, and attempting to evacuate the urine or the stools, performed the operation, on the fourth or fifth day after her admission.

A female sound was introduced into the urethra, and along it the "double lithotome" was carried, as far as the stone which was felt by the instrument. The lithotome thus introduced was opened to No. 10, and drawn out in the usual direction, when the forceps were passed through the wound into the bladder, and efforts made to lay hold of the stone. These, however, were ineffectual, for none could be felt; and the finger was therefore had recourse to, and discovered the existence of a kind of partition, dividing the bladder into two cavities which communicated with each other by a perforation in the centre. The lithotome was now introduced anew, carried into the opening in the central partition, opened to No. 14, and again withdrawn. The finger could now be passed with facility into both the cavities of the bladder, and fragments of stone were seized with the forceps and extracted. Several introductions of the latter instrument were required, and afterwards the bladder was syringed out. The patient was removed to her bed, the pain soon subsided, and the next day she was allowed to return to her friends, at her own particular desire.

Fragments and gravel, mixed with mucus and a little blood, flowed for some time with the urine, but, after the expiration of a month, nothing but a partial incontinence of urine remained. It is now a year or more since the operation, and the child has had no return of her complaint.

III. SPONTANEOUS LUXATION OF THE CERVICAL VERTEBRÆ, ATTENDED WITH HEMIPLEGIA,—CURE.—Veret, a shoemaker, 17 years of age, was received into the Hôtel Dieu on the 12th of October, 1827. The head was much bent, and the chin approximated to the fore-part of the neck; rotation of the head could not be performed, and its flexion and extension were extremely limited and painful; the left side of the body was completely paralyzed, the arm hanging loose by the side of the trunk, and the leg dragging after its fellow in walking. The back of the neck was generally swollen, but opposite the fourth and fifth cervical vertebræ, the swelling was more marked on the left side than the right; the spinous processes were with difficulty felt throughout the neck; the whole was the seat of continual pain; the skin was unaffected. It appeared from the patient's report, that after having slept, during the Summer, in a damp situation, he began to feel pains in the joints, which settled at last in the neck, and became almost constant. Two months afterwards the left side of the body grew weaker than the right, and the loss of power daily increased. In the month of July the neck had become stiff, and shortly afterwards the swelling and deformity took place. Leeches to the neck and baths had been employed, but without the slightest relief to the symptoms. His health was unaffected, the appetite and digestion being good, and the pulse quite natural.

The treatment after his admission consisted in the application of 20 or 30 leeches, repeated at six different times, to the swelling in the neck. The effect of this local depletion was relief to the pain, and a manifest improvement in the properties of motion and sensation. On the 15th Nov. two moxas were applied to the side of the neck, and in ten days afterwards the sloughs came away, and suppuration was promoted by means of issue-beans. The amendment now made rapid strides, and the patient was able, at the end of November, to carry his hand to his head. About this time an abscess formed in the nucha, was opened, and gave issue to a small quantity of pus. In January, the sensibility was nearly as great on the left side as the right, the patient could walk with ease, and every day brought a fresh accession of strength to the limbs. The neck, however, was still stiff, rotation difficult, and a good deal of tumefaction yet remained. Suppuration was kept up till the 15th of April, and in May the patient was dismissed from the Hôtel Dieu, cured.

Whether this was a case of incipient luxation of the vertebræ of the neck or not, it is one of much importance in a practical view. A patient presenting such symptoms as were shewn by the above is generally considered in a very precarious situation indeed, and yet, as far as our own experience goes, he will frequently do well. We remember the case of a scrofulous-looking young man, who was thought by an eminent surgeon to labour under disease of the atlas, or dentata, or upper cervical vertebræ, and on whom a very gloomy prognosis was pronounced. He recovered, we will not say completely, but we frequently meet him walking in the streets, apparently as free from disease as ourselves. The following is a very remarkable case.

James Goddard, æt. 15, was admitted into St. George's Hospital, January 16th, 1827, under the care of Mr. Keate.

The head was inclined downwards to the right side, giving the appearance of the "wry neck," but the muscles were not preternaturally contracted. The patient was unable to rotate the head, or at least what he did was evidently through the medium of the lower cervical vertebræ; he was equally unable to perform the nodding motions of the head upon the atlas, though some power of flexion remained in the cervical vertebræ generally. He complained of pain in the back of the neck, which was worse at nights and aggravated by motion, especially sudden motion, as walking quickly, running, or rising abruptly from his seat, on any of which occasions he was forced to support the chin and the back of the head by his hands. On pressing the spinous processes of the dentata and the third and fourth cervical vertebræ, pain was produced, and on each side of these, especially the left, an evident fulness was perceived. On looking into the mouth, the uvula and velum pendulum palati appeared to be drawn into a line with the hard palate, and to be situated close upon the bodies of the vertebræ. There was no paralysis or numbness; the patient looked scrofulous; the tongue was whitish; the pulse was quick. The patient was a pot-boy, and accustomed to sling the pots over his shoulder, but he never experienced any blow upon the neck. Three months before his admission, he noticed a "pimple" at the back of the neck, which broke, was poulticed, and healed in about three weeks. Eleven weeks exactly before his admission, he began to be affected with a dull aching pain at the back of the neck, increased upon motion. Nothing was done, and the pain got worse, whilst stiffness of the neck supervened. For the fortnight prior to his entrance, he was totally prevented from pursuing his usual avocations.

The treatment consisted in the application of leeches to the back of the neck, followed by repeated blisters. On the 25th of January, an antimonial plaster was applied, and on the 10th of March the following report was taken. He performs the nodding motions of the head with considerable freedom, and rotates it much more boldly than he did. The *wryness* of the head, and prominence of the cervical vertebræ, is diminished—there is no pain on pressing the back of the neck, or forcing the head down upon the atlas—the health is decidedly improved, and the lad has perceptibly gained flesh. The blisters or antimonial plaster were alternately repeated till the second of May, when the last was applied. An emplastrum ammoniaci c. hydrarg. was subsequently employed, and, on the 28th of May, he left the house, being free from all pain, and possessing the power of moving the head with considerable ease.

We remember well, that when we first saw this boy, we drew, in our own mind, but a gloomy prognosis of his case, considering it ulceration of the cartilages, or disease of the bony structure of the upper cervical vertebræ. Whether such disease did actually exist, is more than we can say; but certain it is, that we witnessed his recovery with feelings of pleasurable surprise.

IV. Latent Tubercular Phthisis—Pneumo-Thorax.—A young man was received into the Hôtel Dieu, on the 28th July, 1828, having been affected for three months previously with cough and diarrhœa. He had never spat any blood—and, when he came into hospital, the symptoms were those of chronic enteritis. He coughed very little—expectoration was trifling, and without any purulent character. He was put on a mild soothing regimen, with tepid baths and anodynes; and after more than two months' sojourn in the hospital, he found himself so well as to demand his discharge. This was complied with; but in the evening preceding the day of dismissal, he was taken suddenly with a most violent pain, extending from the left flank to the præcordial region of the same side. This pain was so intense, and its seat so defined, that the physicians immediately concluded that perforation of one of the intestines had taken place, with extravasation of its contents. There was no tenderness of the abdomen, however, on pressure, which caused some doubts as to the existence of so serious an accident. The pain continued during the next day without aggravation, and now the chest was examined. There was but a very feeble respiration heard in the left lung, though the sound on percussion was clear on both sides. In the right lung the respiration was very loud—puerile. The naked ear being applied to the left side of the chest, the metallic tinkling was heard from time to time. On using the Hippocratic succussion, the collision of two fluids of unequal density was heard. The pulse was frequent—decubitus more easy on the right than on the left side. Pneumo-thorax was now evident. In the course of a few days, the symptoms became somewhat mitigated, and the patient was able to walk about the ward. The left side of the chest was bulged out. A week afterwards he died instantaneously, after getting up and taking some breakfast.

*Dissection.* On opening the right side of the chest, the heart was found in that side, opposite the sixth rib. The inferior lobes of the right lung were sound—the superior lobe was sprinkled with tubercles, some of them softened down. When the left cavity of the pleura was opened, a rush of gas come forth, without any particular smell—the lung was compressed against the spinal column, and covered with a layer of whitish coagulable lymph. There was a considerable effusion of clear serum in this side of the chest, containing numerous shreds of coagulated lymph. A blow-pipe was then introduced into the trachea, and air thrown into the lungs, which air soon made its way through two apertures in the pulmonary tissue, and bubbled up through the water effused in the chest. Here, then, was the cause of the pneumo-thorax. There were some distinct ulcerations in the mucous membrane of the ileum. The ascertainment of so serious a malady would have been a thing impossible a few years ago, before auscultation and percussion came to our aid in diagnosis.—*Journ. Hebdomad.*

*Remarks.* Scarcely a journal is now published without the details of some case of pneumo-thorax; hence we may presume that it is by no means an extremely rare disease. We need not offer any particular comments on the foregoing case, since our readers will find the subject fully discussed in the present number of the Journal. We may be permitted to say that an operation would have been justifiable in the above case, and that it would, in all probability, have prevented the fatal termination.

### XXXVIII.

### HOTEL DIEU.

### I. Varieties of Hydrocele—Modifications of the usual Operation.

The operation of injection for hydrocele is certainly, in the great majority of cases, extremely easy and safe in its performance, as well as successful in its results. Cases, however, occasionally occur, where untoward symptoms arise to thwart the objects of the surgeon, or even endanger the patient's existence. Hæmorrhage, either externally, or into the tunica vaginalis, is not very unfrequent, and tetanus has, in several instances, followed the operation. M. Dupuytren, who, from his situation of "chef des travaux anatomiques," has dissected a considerable number of hydroceles, believes that these accidents depend on a variation in the anatomical relations of the hydrocele, a variation of which the surgeon should be aware. The following case is adduced in illustration of the Baron's remarks.

*Case.* Auguste * * *, ætatis 13, of delicate constitution, was admitted into the Hôtel Dieu, with a hydrocele of six months' standing in the left side. The whole circumference of the hydrocele might be grasped without pain, but the instant the upper part was touched, the patient experienced the sensation of the testicle having been meddled with. On using the lighted taper, the testicle was found to be lodged in this part; and, "from the extremity of one of its diameters," the cord passed in front of the scrotum to gain the inguinal canal. In short, the testicle occupied the summit of the hydrocele, and the spermatic cord was situated along its anterior part. Under these circumstances, M. Dupuytren performed the operation at the middle and internal part of the scrotum, in order to avoid the lesion of the organs alluded to above.

M. Dupuytren believes that, had the usual operation been resorted to here, and the testicle and cord been wounded, as they must, it is not improbable that serious mischief might have followed. At all events, he recommends, and we think very justly, that prior to thrusting in the trocar, the position of the testicle and spermatic chord should be carefully ascertained.

### II. Suicidal Amputation of the Penis and Wounds of the Heart.

A considerable number of cases of voluntary mutilation of the genital organs would seem to be occurring in Paris. Out of many such detailed in the Clinique we select the following, as interesting in more than one point of view.

*Case.* A stout dark-complexioned man, about forty years of age, reduced to distress by misfortunes and misconduct, was determined to destroy himself, and had been on the point of throwing himself into the Seine, when, for some misdemeanour, he was put in prison. Believing that he could never re-enter society, he cut off the penis close to the pubes, and, although the knife was extremely bad, amputated the organ in a very scientific and surgeon-like manner. The hæmorrhage was not very great, but, as soon as the circumstance was discovered, he was taken to the Hôtel Dieu, where ligatures were applied on the arteries of the dorsum penis, of the corpora cavernosa, and the bulb; a sound introduced into the urethra; and the wound simply dressed. The patient at this time was tranquil, but a strict surveillance over his motions was practised, as the calm, it was thought, might be delusive. For some days no untoward symptoms occurred, but then there supervened a violent delirium, for which he was bled from the foot, had opiate injections, and gentle purges. The delirium continued, symptoms of depression came on, the urine became loaded with muco-purulent matter, the pulse was in general slow, the extremities cold. The patient died at length in a sub-apoplectic condition three weeks after the infliction of the wound.

*Sectio Cadaveris.* The membranes of the brain were gorged with blood and "serosity," the brain much injected, and its substance very firm. On raising the sternum, a large ecchymosis was found on the pericardium, and the cavity of this serous membrane was half filled with liquid blood. On looking for the source from which this effusion could have come, several small wounds, plugged by a black but fibrinous clot, were discovered on the anterior surface of the ventricles. In the centre of the ecchymosis before described, on the upper and anterior portion of the pericardium, two penetrating narrow wounds were found, and these parts were covered by false membranes. Examination of the anterior wall of the thorax, disclosed between the cartilages of the second and third rib on the left side, a small, nearly round, and cicatrized wound. Beneath the skin, between the intercostal muscles, and beneath the pleura, was a large ecchymosis. The opening in the pleura was marked by a reddish brown spot, surrounded by false membranes. The lung was not wounded, but the wounds in the heart were five or six in number, and principally seated in the right ventricle. The substance of the heart itself was pale and easily torn. The whole of the mucous membrane of the stomach and intestines was chronically inflamed, and several old ulcerations were found in the neighbourhood of the ileo-cæcal valve. The mucous membrane of the bladder also bore marks of chronic inflammation, and the urine contained in the viscus was purulent.

On making inquiries it was found that, after having mutilated himself in prison, not only the knife with which he had committed the act, but also a long needle made use of in the business he followed, (a saddler,) had been taken from his person. This must have been the instrument with which he had made the wounds in the heart, as, after his reception in the Hôtel Dieu, nothing of the kind could have come within his reach, and indeed the idea was confirmed by the state of the cicatrix externally. The wounds thus inflicted were in fact little more than a species of acupuncturation, which will readily account for the trifling hæmorrhage and almost total absence of symptoms indicative of their existence during life. The pulse, as was stated in the history of the case, was slow, the action of the heart was apparently undisturbed, there was no dyspnœa, nor any syncope. Whether, in the event of the patient's having lived, these wounds in the muscular substance of the heart, would have caused any serious symptoms is more than any one can absolutely determine. For our own parts, we should feel great difficulty in conceiving that the pericardium could remain half filled with blood, without producing, sooner or later, some disturbance in the system.

III. Aneurism of the Brachial Artery after Bleeding—Operation of tying the Vessel above—M. Dupuytren embarrassed.

To a man whose learning is only of the schools, or who having very little of any description, sits down in his closet or his garret to criticize or calumniate those who are unfortunate enough to be his brethren, nothing would seem easier than the operation of securing the brachial artery. In the dissecting room, frequently indeed to a dexterous surgeon on the living body, a touch or two of the knife is almost sufficient to expose the vessel, and the rest is sufficiently simple. Occasionally, however, and no one beforehand can foresee the difficulties; occasionally, we say, even the operation of tying the brachial artery is tedious, difficult, and unfortunate, in the hands of the best and most dexterous operator. The following case will illustrate the truth of our remarks.

*Case*. Jean Auguste, ætatis 32, a hawker, of strong constitution, was bled from the arm, and received a wound in the brachial artery during this operation. The hæmorrhage which ensued was stopped by pressure, but returned at the end of some hours, and produced a considerable extravasation of blood in the fore-arm. This, which constituted a diffused aneurism, at the end of some time, by the partial absorption of the blood, and conversion of the surrounding cellular membrane into a species of sac, formed a circumscribed one, and six weeks after the accident, when the patient was received in the Hôtel Dieu, the tumour was no larger than a hen's egg. On examining it with care, the pulsations were found to be obscure, and ceased altogether on forcible extension of the arm, as well as on compressing the brachial artery above. From these phenomena it was thought, indeed it was clear, that the opening from the artery into the sac was either very small or oblique, and also that the latter received its blood from only one source.

Under these circumstances, the operation of securing the artery in the middle of the arm was performed by M. Dupuytren. It was cut down upon, exposed at the inner border of the biceps, and separated from the median nerve in the course of a very few minutes; but now, from the pain, or the cold, or other causes that "we know not of," the heart began to beat but feebly, the pulsations in the artery ceased, it could not be distinguished from the nerve, the surgeon was embarrassed, and, finally, upwards of half an hour elapsed before this apparently simple operation was completed. The patient by this time had rallied, and the artery pulsated so plainly, that the doubts which had previously existed were dispelled.

The reporter observes, that the amphitheatre of the Hôtel Dieu is so constructed, that in order to allow the spectators to see the operation, M. Dupuytren was obliged to stand *behind* the limb, which was necessarily extended horizontally, instead of being laid in an easy supine position. These circumstances were much against the surgeon, and no doubt added to the difficulties met with. In the evening the limb was of its natural temperature, and next morning the pulse was felt in the radial artery. An erysipelatous blush had attacked the wound, accompanied with fever, and, after some time, the cutaneous inflammation extended to the fore-arm. These untoward symptoms, however, disappeared under the administration of diluents, &c. and on the 4th day, the pulsations in the radial artery were felt no more—the tumour was still—the limb of its natural warmth, and entirely free from pain. The circulation became re-established, though slowly, below the ligature, but several attacks of erysipelas succeeded on the arm and fore-arm, and were repelled by the application of a blister to the centre of the inflamed part. Gastric symptoms also arose, and required two bleedings from the arm, and some alteratives. The aneurismal tumour quickly softened, the cicatrix of the lancet-wound gave way, three or four ounces of black, liquid, inodorous blood issued from it, an abscess followed, but, finally, the patient recovered completely, and at the time of closing the report, was daily expected to leave the hospital.

M. Dupuytren saw this softening of the contents of the sac in a case of axillary aneurism, after the application of a ligature on the subclavian. In that instance he opened the sac by an incision. In a case of aneurism of the femoral artery, we witnessed this circumstance after the operation of tying the vessel, and the patient sank under the irritation excited by the dissolution and putrescence of the contents of the sac. M. Dupuytren attributed the softening of the blood, &c. to the erysipelas which occurred in both his patients, but in that to which we have alluded, no erysipelatous inflammation, we believe, took place.

### LVIII.

### HOTEL DIEU.

### I. POLYPI AND FOREIGN BODIES IN THE EXTERNAL MEATUS AUDITORIUS.

To remove the foreign bodies so frequently lodged in the meatus auditorius, and which sometimes foil the best directed efforts to dislodge them, M. Dupuytren uses the following instrument. It consists of a small pair of forceps, the branches of which are twice bent at a right angle, so that the hand, when it grasps them, is prevented from obstructing the view of the surgeon into the meatus. The branches terminate in two kinds of spoons of a rounded form, pierced with numerous holes, and armed with rough teeth. The joint is about an inch from the lower extremities of the branches, which confers sufficient power on the instrument for seizing the bodies it is wished to extract. With these little forceps the reporter assures us that M. Dupuytren is very successful.

### II. ANGINA PECTORIS—CORONARY ARTERIES PERFECTLY SOUND.

Scarce a month elapses, scarce a medical society meets where the subject of angina pectoris is broached, that we do not either read or hear it considered to depend on ossification of the coronary arteries as a matter of course. If some individuals, as some there be, will not listen to facts, it is their fault, not ours. It is not a little singular, that although the said ossification is considered by many in this country as the whole and sole cause of angina pectoris, M. Recamier, placed at the head of the Hôtel Dieu, is said to have never witnessed an instance of this morbid change in the bodies of those who have died of the disease. M. Recamier, indeed, who has witnessed a considerable number of cases, rejects in toto the idea of its depending on this or that organic alteration, but seems, like Laennec, to consider it as a species of neuralgia, frequently present with various structural lesions of the heart, but equally present without them. The dissection we are now to relate will serve, at any rate, to shew that the disease may prove fatal, without the slightest appreciable lesion of the coronary arteries.

Josephine Vala, 34 years of age, had been subject, for three or four months, to symptoms of angina pectoris, when she entered the Hôtel Dieu, under the care of M. Recamier. This was on the 14th of November, and during the night of the 21st she suddenly awoke, vomited once or twice, heaved a groan or two, and suddenly expired. On dissection, the veins of the brain were found turgid, the pia mater and ventricles were infiltrated with serum, the medullary substance was firm and presented black spots, the cerebellum did the same. The heart presented an "eccentric hypertrophy" of its left ventricle, its serous envelopes were sound, *so were the coronary arteries*, the cavities in the right side were perfectly natural. On the left, the texture of the organ was firm, but its internal half had a lighter tint than its outer. The mitral valve was in a healthy state, the sigmoid were slightly thickened. The internal coat of the aorta was destroyed throughout its greatest part, the middle coat presented a number of irregular yellow spots, separated by intervals of a white pearly tint. In many places the débris of the internal coat were hanging loosely from the middle. These appearances were most distinct in the thoracic aorta, but decreased as the tube proceeded from its origin. The uterus was a good deal larger than it should be, gorged with blood, and deeply coloured of a reddish brown, whilst its cavity contained a jelly-like matter. Other than these no appearances of any import were observed.

To the tail of the case four propositions of M. Recamier are added, which, although they cannot justly be said to contain all the sting of the subject, are yet, we believe, extremely important. The doctor observes first, that the same symptoms are not always the expression of the same cadaveric alteration; secondly, that in practice the language of symptoms is even more important than the language of pathology; thirdly, that a given symptom is no surer a test of the lesion which produces it, than the view post-mortem of a structural alteration would indicate the symptom during life which it produced; and, lastly, that a sensible physician would neither neglect the evidence of symptoms, nor that of dissection.

### III. OPERATION FOR FEMORAL HERNIA —ARTIFICIAL ANUS—CURE.

An old woman, in indifferent health, and who had worked hard to gain her bread, had been subject for about eight months to reducible femoral hernia, for which she had never worn a bandage or truss, when on the 26th of last November she was attacked with colic and constipation. Nausea followed, and in the night there was vomiting of bilious and fetid matters, which continued till the 28th, when she entered the Hôtel Dieu. M. Sanson, who saw the patient, immediately recognised a femoral hernia, and, after an unsuccessful attempt at reduction, proposed the operation, which the patient refused. Next morning M. Dupuytren obtained her consent, and proceeded to operate by making, over the tumour, a vertical incision two inches long through the skin. The lamellated and adipose tissue beneath was cautiously divided, and after separating some lymphatic glands, the surgeon arrived at the sac, which was very elastic and hard, of a violet colour, and about the volume of a nut. The sac was carefully opened with the bistoury, when a small jet of limpid and colourless liquid took place. The opening was enlarged, and the finger introduced, when the internal lining membrane was felt to be villous, and nothing could be found contained within, in short, it was discovered that the finger was not in a hernial sac, but absolutely in the cavity of the gut, the two having contracted inseparable adhesions. It was also ascertained by the finger's passing up through the crural ring that the latter was not the seat of stricture.

A female catheter was inserted into the intestine, and directed under the crural arch, when some drops of a mucous inodorous matter were discharged. The instrument was a little withdrawn and carried in another direction, when a gush of yellow liquid fæces issued in quantity sufficient to fill two large basins. The catheter was left in and fæcal matters flowed during the day in great abundance, whilst the urgent symptoms speedily subsided. The wound was lightly dressed, and in five or six days gas escaped from the anus, mucous and fæcal matters succeeded under the use of laxative lavements, and before the expiration of a month, the artificial anus, by means of steady compression, gave issue to no more stercoraceous substance. The wound contracted, and, on the thirty-eighth day after the operation, scarcely the slightest fistulous opening remained.

As the reporter of the case in the Clinique observes, it is not a little irregular that a hernia which was perfectly reducible till three days before the operation, should yet at that time have presented such intimate adhesions betwixt the sac and the surface of the gut. It is clear, nor is it attempted to be concealed, that M. Dupuytren opened the gut by mistake, but it comes to be a question whether the proceeding thus undesignedly adopted was not, under existing circumstances, the proper one. If the adhesions between the sac and the intestine be so firm that no fluid whatever is effused between them, and an intimate union has ensued, we cannot, for our own parts, conceive that any alternative remains to the surgeon but that of laying open the gut. It would surely be the height of impropriety to return the sac and gut together, even supposing that it could be done, and quite as dangerous to allow them to remain in statu quo. We believe then, and the case itself strengthens the opinion as far as one case can go, that the practice, although unintentional, was justified, and called for by the actual condition of the parts.

### IV. VARIX OF THE SAPHENA COUNTERFEITING FEMORAL HERNIA.

A joiner, thirty-two years of age, who seven years before had the left saphena major tied by M. Dupuytren above the ankle and below the knee for a varicose state of the vein in the interval, presented himself with a return of the varices in the right leg. The right saphena was dilated up to its termination in the femoral vein, at which situation was a tumour presenting not a few of the characters of femoral hernia. It was roundish in shape, unequal on the surface, elastic, and removable by firm and long-continued pressure. On compressing it, a feeling of crepitus or gurgling was occasionally given to the finger, its size was that of a small egg, it was not at all painful, enlarged a little on coughing, and speedily regained its former volume, when the pressure by which it had been diminished was removed. The position, the volume, and the form of the tumour, as well as its increasing in size upon coughing, were features of resemblance to femoral omental hernia. However, on compressing strongly the groin on the ramus of the pubes, so as to compress entirely the crural ring through which the femoral hernia passes, the volume of the tumour continued undiminished, and besides, the tumour received a pulsation from its contiguity with the femoral artery, a circumstance which would scarcely occur to a femoral hernia.

On these accounts M. Dupuytren experienced no great difficulty in deciding that the tumour was a varix of the saphena at its junction with the femoral vein, immediately under the crescentic arch of the fascia lata. Sir Astley Cooper observes in his lectures that he has seen a case of enlargement of the femoral vein, "which had somewhat the appearance of a femoral hernia, but was readily detected by pressing on the iliac vein above, whilst the patient was recumbent, when the tumour immediately appeared." This was an item in the diagnosis which does not appear to have entered into M. Dupuytren's experiments. We have reason to believe, that cases have occurred, where the error of considering a varicose condition of the femoral or saphena veins for a hernia has been made, and a surgeon even called in for operation.

